# A comparative study on the hepatoprotective action of bear bile and coptidis rhizoma aqueous extract on experimental liver fibrosis in rats

**DOI:** 10.1186/1472-6882-12-239

**Published:** 2012-11-29

**Authors:** Ning Wang, Yibin Feng, Fan Cheung, Oi-Yee Chow, Xuanbin Wang, Weiwei Su, Yao Tong

**Affiliations:** 1 School of Chinese Medicine, The University of Hong Kong, Pokfulam, 10 Sassoon, Road, Hong Kong, P. R. China; 2 School of Life Science, Sun Yat-Sen University, 135 Xingangxin Road, Haizhu District, Guangzhou, 510275, P. R. China; 3 Laboratory of Chinese Herbal Pharmacology, Renmin Hospital, Hubei University of Medicine, Shiyan, 442000, P. R. China; 4 School of Pharmacy|, Hubei University of Medicine, Shiyan, 442000, P. R. China

**Keywords:** Coptidis rhizoma, Bear bile, Liver fibrosis, Alternative, Comparative study

## Abstract

**Aim of the study:**

Bear bile and Coptidis Rhizoma have been used in Chinese medicine with a long tradition in treating heat-diseases. Both bear bile and Coptidis Rhizoma are used to treat liver diseases in clinical practice of Chinese Medicine. Since bears are currently endangered, it raises the question whether the use of bear bile is ethical. To look for substitute for bear bile, the aim of this study is to compare the anti-fibrotic effects of Coptidis Rhizoma and its major component berberine with the actions of bear bile and its major compound tauroursodeoxycholic acid on experimental liver fibrosis in rats.

**Method:**

Quality assessment was conducted with high performance liquid chromatography. The experimental liver fibrosis in rats was induced by carbon tetrachloride, alcohol, and bile duct ligation respectively. The biochemical criteria in the blood and tissue samples were measured to evaluate the anti-fibrotic properties and underlying mechanisms of the drugs.

**Results:**

Coptidis Rhizoma Aqueous Extract (CRAE), berberine, and bear bile exerted anti-fibrotic properties on various liver fibrosis models in rats. CRAE and berberine significantly reduced the peroxidative stress in liver through increasing the superoxide dismutase enzyme activity. CRAE and berberine were able to excrete bilirubin products from the liver and protect hepatocytes from cholestatic damage. The effect of CRAE and berberine are comparable to that of bear bile.

**Conclusion:**

Instead of using bear bile, CRAE and berberine can be potential substitutes in treating liver fibrosis.

## Background

Bear bile can be obtained from the gall bladder of brown bear (*Ursus arctos*) and Asiatic black bear (*Selenarctos thibetanus*). The creature must be either sacrificed, or inserted a metal catheter into its gall bladder extract the bile. Currently in China, there are no legislatures to prohibit the establishment of bear farming to obtain bile. Many reports concluded that bear farming should be abolished due to the inhumane treatment of the creatures. [1]. Brown bear and Asiatic black bear are listed in Convention on International Trade in Endangered Species of Wild Fauna and Flora (CITES) Appendix I. The use of bear bile in traditional Chinese medicine conflicts with the morals of animal protection [2]. Based on the reasons above, it is necessary for the science community to design systematic, serious and comparative research to get convincing data for substitute for bear bile.

There are only four sources of alternatives to bear bile being reported currently. These include bile from other animals, derivatives from plants, chemical compounds, and synthetic bear bile. Recently, our research provides evidence that bear bile can be easily replaced by those of other animals for certain situations [3]. Despite being an effective option, the sacrifice of animals is frowned upon by animal rights activist. The replacement of bear bile through the use of herbs and plants provides a means to treat liver fibrosis without harming any animals. However, there are some whom remain dubious about using alternatives and hold true to the use of bear bile. It should be an arduous task to replace the bear bile by product with the same composition, but it is possible to evidence the comparable therapeutic effect of herbal substitute with scientific approaches. As of today, derivatives from plant remain as only suggestions and not proven treatments. The use of Coptidis Rhizoma may be suggested because of its similarity with bear bile in the traditional use according to Chinese Medicine theories, and both Coptidis Rhizoma and bear bile have similar clinical indications and scientific evidences in treating liver diseases [4-9]. However, there is no study reporting the comparative study on the anti-fibrotic action of Coptidis Rhizoma and bear bile and their underlying mechanism.

Liver fibrosis is an abnormal wound-healing process in which the connective tissues proliferate under certain conditions. Several kinds of continuous and chronic liver injuries could induce liver fibrosis, among which Hepatitis B viral infection (HBV) is the main cause of liver fibrosis in China, while hepatitis C infection and alcoholic intoxication cause liver fibrosis in the United States, Europe and Japan [4]. Previous studies on the pathogenesis of liver fibrosis indicated that activation of hepatic stellate cells, over-expression and over-accumulation of extracellular matrix (ECM) protein, and collagen-like protein in liver tissue may be involved [10]. The treatment of liver fibrosis includes eliminating the primary disease, modulating the immune system, suppressing liver inflammation, inhibiting the accumulation of ECM protein, and reducing complications [11].

In this study, we aimed to examine the anti-fibrotic effect of Coptidis Rhizoma (CR) and its major component berberine, and compare their pharmacological action with that of bear bile (BB) and its major compound tauroursodeoxycholic acid (TUDCA). Quality assessment of CR aqueous extract (CRAE) and BB were performed using HPLC-DAD methods. Three animal models were established for testing relevant doses of CRAE, berberine, BB and TUDCA, which is obtained from dose screening of testing drugs. CCl_4_, Bile duct-ligation (BDL) and alcohol-fed were introduced to induce experimental liver fibrosis in rats. Serum aspartate aminotransferase (AST), alanine transaminase (ALT), and total bilirubin (TBil) were measured to determine the hepatoprotective effect of CRAE, berberine, BB, and TUDCA. Tissue Hydroxyproline (HyP) content was examined to determine the fibrosis level in liver. Tissue superoxide dismutase (SOD) was determined to elucidate of underlying mechanism of the drugs. Both negative control and positive controls (Silymarin) were used in the experiments. We found both CRAE and berberine significantly attenuated liver fibrosis in rats as well as BB, but not TUDCA. Mechanism studies showed that CRAE and berberine selectively increased the SOD activity and promoted bilious product excretion, which reduced the oxidative stress of the hepatocytes. Our study reveals that CRAE and berberine may be the possible alternatives to bear bile.

## Materials and methods

### Chemicals and drugs

Tauroursodeoxycholic acid (TUDCA), Silymarin, Berberine (BBR) and Carbon tetrachloride (CCl_4_) were purchased from Sigma-aldrich (USA); Coptidis Rhizoma (CR, *Huanglian* in Chinese) was obtained from the Chinese Medicine Good Agriculture Practice (GAP) base for *Huanglian* in Chongqing, China. The Bear bile (BB) obtained from Asiatic Black Bear was purchased from Hang Hing Company (Hong Kong, license No. APO/PL 2384/07).

### Sample preparation

To prepare the CR aqueous extract (CRAE), raw materials were cut into small pieces and 50 grams of crude CR was boiled in 500 mL of distilled C for 1 hour and then filtered. The filtrate was then evaporated to dryness and the dry extract powder was collected and stored at −20°C until used.

### Chemical analysis

High Performance Liquid Chromatography (HPLC) with Photodiode Array (PDA) Detector was conducted to determine the chemical constitution of CRAE and BB. To assess the chemical composition of CRAE with conditions as literature reports [5,6] with some modifications, 10 μL of CRAE (0.5 mg/mL in methanol) was injected and eluted through RP-C_18_ column (250 mm x 4.0 mm, 5 μm, Alltech, USA) with mobile phase as acetonitrile-25 mM potassium dihydrogen phosphate (25:75). The flow rate was 1.0 mL/min and detection was performed at 350 nm. An equal volume of berberine in methanol was injected as standard marker. The conjugated bile acids was assessed using A Nova-Pack® C18 column (300 mm × 3.9 mm I.D., particle size, 4 μm; Waters, USA) as solid phase and MeOH-25 mM potassium dihydrogen phosphate (65:35) as mobile phase. The column was kept at 40°C. The flow rate and detection wavelength is 0.9 mL/min and 200 nm. Sodium taurouesodeoxycholate (TUDCA) was injected as standard marker.

### Animals

Male KM mice weighing 20 ~ 25 g and male SD rats weighing 220 ~ 250 g were kept under conditions of controlled temperature (25 + 2 °C) and illumination (12 h light cycle starting at 06:00 AM). Animals were maintained on laboratory foodstuff and water freely. All experiments were complied with the international and Chinese guidelines and approved by the regional ethics committee.

### Acute liver damage

The acute liver damage mice model was established by intraperitoneally injecting 3 mg/kg of CCl_4_ (1:1 mixed with olive oil) half hour before drug treatment.

### CCl_4_-induced liver fibrosis

Liver fibrosis rat model was established by administration of CCl_4_. Animals were grouped, and all except the normal group received 3 mg/kg of CCl_4_ (1:1 mixed with olive oil) intraperitoneally twice per week for seven weeks. Rats in the normal group received 3 mg/kg of olive oil subcutaneously during the same period.

### Bile duct ligation (BDL)-induced liver fibrosis

Extrahepatic cholestasis was produced by common bile duct ligation (BDL). In brief, under ether anesthesia, the common bile duct was ligated with 3–0 silk and sectioned between the ligatures. The midline abdominal incision was closed with catgut. Sham-operated rats had their bile duct exposed but not ligated or sectioned. All rats were caged at 24°C with a 12:12 h light–dark cycle, and were allowed free access to food and water for two days before the study. All ligated rats were then divided into six groups. The induction group (BDL rats), normal group, and sham group was administrated equal volume of PBS during the period of experiment.

### Alcohol fed-induced liver fibrosis

The alcohol fed-induced liver fibrosis model in rats was established as according to the literature with some modifications [12]. Briefly, rats in each group, except for normal group, received a mixture of ethanolpyrazole-corn oil (10 mL-25 mg-2 mL) twice every day through oral gavage (5 mL/kg body weight) for seven weeks. During the same period, animals received respective daily treatment orally. In the normal group, rats were given equal volumes of PBS every day.

#### ***Treatment***

Optimal doses of each drug for the treatment of liver fibrosis in animal model were determined based on the results of acute liver damage model. Rats in normal, shamed, and model group received 10 mL/kg of distilled water per day by oral administration, as well as 120 mg/kg berberine, 600 mg/kg CRAE, 270 mg/kg BB or 20 mg/kg TUDCA, respectively. 150 mg/kg of Silymarin was administrated orally to rats as a positive control. All treatments lasted for seven weeks.

### Biochemical analysis

Animals were sacrificed at the end of the experiment by an overdose of pentobarbitone (Phenobarbital 200 mg/kg, i.p) immediately. Blood samples were collected and serum aspartate aminotransferase (AST), alanine transaminase (ALT) and total bilirubin (TBil) were detected using biochemical auto-analyzer. The results were normalized by the total protein level in serums. Liver samples in rats were collected and the tissue proteins were extracted by homogenization. The Tissue Hydroxyproline (HyP) content and Tissue Superoxide Dismutase (SOD) were measure using commercial detection kits (Jiancheng Bioengineering Institute, Nanjing, China).

### Histological analysis

Livers from rats were removed and fixed in 10% formaldehyde buffer for 24 h. Paraffin sections were prepared and cut into 5 μm thick sections. Sections were stained with hematoxylin and eosin staining (H&E staining). To determine the liver injury, we use semi-quantitative method according to our previous publication and three individual professional pathologists were invited [5]. A combinational score was provided to each section based on histologist’s judgments on the grade of severe hepatocyte cell death, inflammatory cell infiltration and fibrosis. To further identify the level of fibrosis, five phases of liver fibrosis were defined according to the literature. The S0 phase showed no signs of observed fibrosis. The S1 phase showed no extension of portal area fibrosis. The S2 phase exhibited fibrosis occurring in the portal area with an intact lobule structure. The S3 phase shows fibrosis associated with a broken lobule structure, but no signs of cirrhosis. The S4 phase shows fibrosis and the formation of cirrhosis. Fibrotic area within 1.5 mm2 of each section was measured under the light microscope. Six sections in each group was randomly sampled and analyzed under microscope.

### Statistic analysis

Data were expressed as Data were expressed as mean ± standard deviation (S.D.) and statistical comparisons were performed using Student-Newman-Keuls test [13] using SPSS 11.5.

## Results

### Quality assessment of CRAE and BB

**Figure 1:**
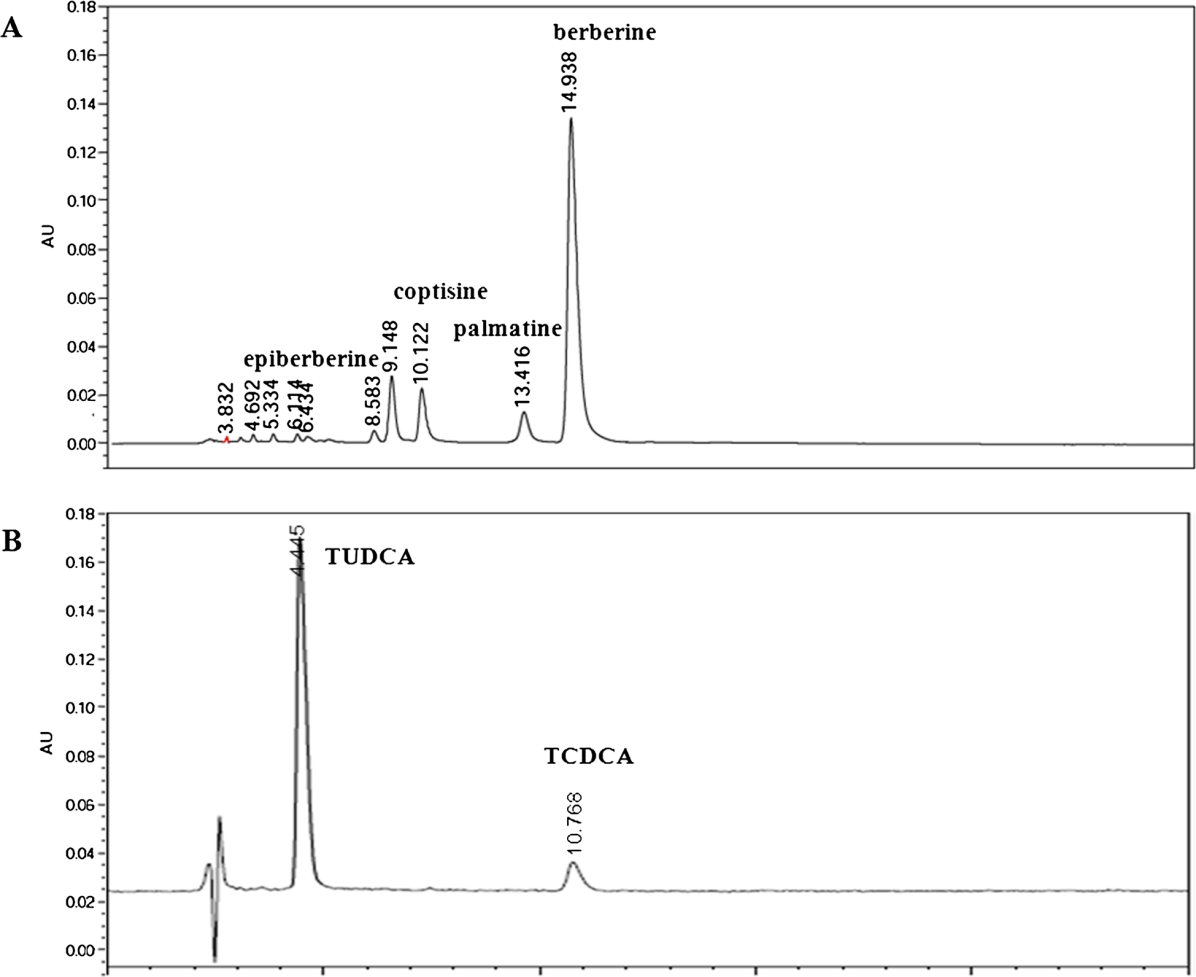
**Quality assessment of CRAE and BB by HPLC-DAD method. **(**A**) shows chromatogram of CRAE. (**B**) shows chromatogram of BB.

To evaluate the quality of CRAE and BB, HPLC-DAD was introduced to quantify the major active compound in CRAE and BB. According to the China Pharmacopeia (Edition 2010), berberine was injected as standard marker of CR, and the yields of epiberberine, palmatine and coptisine were calculated based on the ratio of their peak area values to berberine. The chromatogram of CRAE was shown in Figure 1A and the yields of epiberberine, palmatine, coptisine and berberine were 0.47%, 2.54%, 1.77%and 23.05%, respectively, which was consistent with our previous reports [5,6]. Conjugated bile acids were determined by HPLC-DAD and TUDCA was injected as standard marker to evaluate the quality of BB sample. The result showed that TUDCA was the most abundant component in BB and the yield of TUDCA in BB was 7.84%, which was consistent with our previous report [3].

### Optimization of drugs dose

To better understand the effect of different drug agents, the dose-dependent effect of each drug should be evaluated. CCl_4_-induced acute liver damage in mice was used as animal model to study the dose dependent effect of BB. The results showed that BB had potent effect on reducing the serum’s AST and ALT level. The optimized dose for the effect was about 270 mg/kg (Table 1). Since TUDCA was the major active compound in BB, the dose of TUDCA was determined subject to its proportion in BB. Calculated by HPLC, TUDCA comprises about 7.84% of BB as shown in Figure 1, so the optimized dose of TUDCA was estimated to be about 20 mg/kg. Based on our previous study [5], the optimized dose of CRAE and berberine were 600 mg/kg and 120 mg/kg, respectively.

**Table 1:** **The dose-dependent effect of BB on AST and ALT level in CCl**_**4**_**-induced acute liver toxicity in mice (n = 8,**$\bar{x}\pm s$**)**

**Group**	**N**	**Dose(mg/kg)**	**AST(U/I)**	**ALT(U/I)**
normal	8	—	99.0 ± 15.8	44.6 ± 10.6
CCl_4_	8	—	271.3 ± 142.0^△ △^	209.5 ± 153.9^△ △^
Silymarin	8	100	133.8 ± 49.9 ^**^	80.4 ± 40.5 ^*^
BB	8	10	202.5 ± 205.5	237.8 ± 383.2
BB	8	30	123.8 ± 17.7 ^**^	63.9 ± 49.6 ^**^
BB	8	90	119.0 ± 58.6 ^*^	63.9 ± 37.3 ^**^
BB	8	270	123.8 ± 22.0^**^	45.5 ± 12.0^**^
BB	8	810	127.3 ± 56.4 ^**^	59.3 ± 25.3 ^**^

^△^p < 0.05 when compared with normal group; ^△△^p < 0.01 when compared with normal group; ^*^p < 0.05 when compared with model group; ^**^p < 0.01 when compared with model group.

### Assessment of modeled animal behaviors with treatment of different agents

Frequent assessments were performed to monitor the animal behaviors after treatment of different agents. The body weight, food intake and water drinking were monitored on weekly basis. The results show significant loss of body weight in rats with liver fibrosis induced by either CCl_4_, BDL, alcohol (Tables 2, 3 &4). The drug treatments could not attenuate the body weight loss in modeled rats. However, in alcohol-induced liver fibrotic rats, silymarin restored the body weight significantly (Table 4). There were no significant changes on the weight loss of modeled rats between the treatment of BB and CRAE (p > 0.05). Interestingly, we found that both CRAE and BB could increase water consumption in bile-duct ligation-induced liver fibrotic rats (p < 0.05, Table 3), while there’s no significant effect of the two agents on water drinking in the other two liver fibrosis models (p > 0.05, Tables 2 and 4). Considering that increasing drinking may facilitate the toxic bilirubin excretion, the effect of CRAE and BB on the water consumption may be of benefit for the recovery of liver fibrosis in modeled rats. Neither of the drug treatments had any effect on the food intake of the specimens.

**Table 2:** **Impact of drug intervention on the animal daily behavior in CCl**_**4**_**-induced liver fibrotic rats (n = 7,**$\bar{x}\pm s$**)**

**Index**			**Body weight (g)**				**Water drinking (mL per rat)**				**feeding (g per rat)**			
**Group**	**dose mg/kg**	**n**	**D0**	**D14**	**D35**	**D49**	**D0**	**D14**	**D35**	**D49**	**D0**	**D14**	**D35**	**D49**
Normal	—	7	195 ± 6.1	303 ± 10.0	368 ± 41.2	416 ± 25.4	41.0 ± 1.4	60.0 ± 21.2	43.1 ± 11.5	61.4 ± 0.9	21.8 ± 2.0	37.6 ± 0.6	25.7 ± 0.2	23.8 ± 0.2
Model	—	7	198 ± 9.5	267 ± 40.3^△△^	288 ± 15.6^△△^	302 ± 17.2^△△^	36.7 ± 2.4	41.7 ± 9.4	35.0 ± 4.7	26.8 ± 2.5^△^	18.1 ± 1.2	29.5 ± 2.4	20.2 ± 4.5	17.6 ± 7.4
Silymarin	150	7	192 ± 8.3	260 ± 40.4	293 ± 46.0	306 ± 55.1	47.0 ± 9.9	33.8 ± 5.3	27.5 ± 0.0	22.9 ± 7.7	21.7 ± 2.3	22.3 ± 9.8	17.3 ± 4.1	19.5 ± 5.0
Berberine	120	7	195 ± 5.3	277 ± 23.3	292 ± 33.4	309 ± 28.9	41.0 ± 1.4	37.5 ± 0.0	40.4 ± 4.1	34.3 ± 7.8	19.1 ± 2.9	25.3 ± 0.2	20.1 ± 1.1	19.5 ± 0.5
CRAE	600	7	195 ± 7.5	271 ± 24.6	300 ± 30.8	318 ± 35.7	34.0 ± 8.5	35.6 ± 2.7	34.4 ± 0.9	28.3 ± 4.7	16.1 ± 0.0	25.3 ± 3.3	17.8 ± 4.1	19.3 ± 1.2
Bear bile	200	7	201 ± 1.3	285 ± 21.4	321 ± 19.1	358 ± 40.7	33.0 ± 1.4	31.9 ± 9.7	36.3 ± 7.1	31.2 ± 3.5	19.0 ± 0.6	24.8 ± 3.7	22.5 ± 2.2	19.9 ± 0.3
TUDCA	20	7	194 ± 8.8	278 ± 18.1	296 ± 22.3	320 ± 28.2	41.5 ± 3.5	31.3 ± 8.8	37.1 ± 7.7	26.3 ± 8.8	16.2 ± 6.8	23.7 ± 0.3	15.3 ± 2.1	18.9 ± 2.8

^△^p < 0.05 when compared with normal group; ^△△^p < 0.01 when compared with normal group.

**Table 3:** **Impact of drug intervention on the animal daily behavior in bile duct ligation-induced liver fibrotic rats (n = 7,**$\bar{x}\pm s$**)**

**Index**			**Body weight (g)**				**Water drinking (mL per rat)**				**feeding (g per rat)**			
**group**	**dose mg/kg**	**n**	**D0**	**D14**	**D21**	**D28**	**D0**	**D14**	**D21**	**D28**	**D0**	**D14**	**D21**	**D28**
Normal	—	7	207 ± 12.0	328 ± 9.6	382 ± 12.1	389 ± 17.7	37.5 ± 0.0	56.9 ± 8.0	53.1 ± 2.7	51.3 ± 8.8	32.3 ± 11.1	31.1 ± 18.4	32.8 ± 2.4	33.8 ± 0.7
Sham	—	7	214 ± 5.4	321 ± 18.9	384 ± 26.2	389 ± 28.3	54.4 ± 6.2	60.0 ± 14.1	60.6 ± 4.4	62.5 ± 7.1	38.4 ± 0.7	34.0 ± 3.0	32.0 ± 0.0	27.1 ± 5.1
Model	—	7	213 ± 7.8	290 ± 16.2^△△^	332 ± 18.2^△△^	338 ± 26.0^△△^	41.3 ± 1.8	74.7 ± 1.9	54.0 ± 5.7	48.3 ± 11.8	8.7 ± 3.9^△△^	27.1 ± 1.6	27.7 ± 3.0	26.2 ± 1.9^△△^
Silymarin	150	7	210 ± 6.2	260 ± 24.4	289 ± 15.8	291 ± 15.2	57.4 ± 1.9	85.0 ± 49.5	72.3 ± 0.9*	59.3 ± 3.8*	8.3 ± 2.7	26.9 ± 4.6	22.9 ± 0.4	21.0 ± 0.6
Berberine	120	7	210 ± 6.2	294 ± 29.9	342 ± 31.0	340 ± 32.0	36.1 ± 0.2	76.3 ± 23.0	78.8 ± 3.1^**^	62.5 ± 7.1**	9.4 ± 1.0	25.7 ± 3.9	28.1 ± 1.9	28.9 ± 3.0
CRAE	600	7	208 ± 6.0	275 ± 25.2	320 ± 3.5	324 ± 2.4	55.0 ± 14.1	70.8 ± 8.2	75.8 ± 5.9**	61.3 ± 15.9*	7.8 ± 1.7	21.6 ± 11.0	28.6 ± 3.9	28.7 ± 1.3
Bear bile	200	7	207 ± 7.5	293 ± 38.5	331 ± 60.5	345 ± 64.7	34.4 ± 0.9	81.3 ± 1.8	75.6 ± 2.7**	72.5 ± 10.6**	8.2 ± 2.2	24.6 ± 1.2	28.2 ± 1.9	29.4 ± 0.8
TUDCA	20	7	212 ± 5.5	279 ± 30.1	311 ± 58.9	307 ± 72.5	30.1 ± 5.5	76.7 ± 28.3	78.3 ± 11.8**	63.3 ± 18.9*	6.9 ± 2.6	24.2 ± 1.3	26.3 ± 2.1	24.8 ± 4.6

^△^p < 0.05 when compared with normal group; ^△△^p < 0.01 when compared with normal group; ^*^p < 0.05 when compared with model group; ^**^p < 0.01 when compared with model group.

**Table 4:** **Impact of drug intervention on the animal daily behavior in alcohol fed-induced liver fibrotic rats (n = 7,**$\bar{x}\pm s$**)**

**Index**			**Body weight (g)**				**Water drinking (mL per rat)**				**feeding (g per rat)**			
**Group**	**dose mg/kg**	**n**	**D35**	**D42**	**D49**	**D56**	**D35**	**D42**	**D49**	**D56**	**D35**	**D42**	**D49**	**D56**
Normal	—	7	399 ± 24.8	391 ± 27.6	435 ± 15.4	440 ± 13.9	33.8 ± 8.84	48.1 ± 0.88	42.5 ± 8.84	44.1 ± 0.18	24.5 ± 2.81	30.6 ± 7.78	27.2 ± 2.83	28.5 ± 2.76
Model	—	7	296 ± 33.6^△△^	272 ± 30.1^△△^	293 ± 33.8^△△^	326 ± 49.4^△△^	39.3 ± 5.48	37.0 ± 8.31	37.4 ± 4.40	35.8 ± 15.8	21.8 ± 3.87	17.7 ± 6.74	16.3 ± 6.07	21.7 ± 5.34
Silymarin	150	7	321 ± 33.3	317 ± 47.9^**^	357 ± 45.4^**^	378 ± 43.1^**^	41.3 ± 17.7	49.2 ± 13.0*	55.3 ± 0.47**	56.0 ± 6.66*	24.2 ± 6.08	13.8 ± 8.22	23.4 ± 1.61	25.0 ± 0.08
Berberine	120	7	278 ± 27.1	281 ± 30.7	296 ± 31.3	304 ± 35.8	31.2 ± 5.07	42.5 ± 15.2	34.1 ± 2.79	29.8 ± 3.06	18.0 ± 6.28	14.6 ± 2.31	19.0 ± 6.15	18.1 ± 3.20
CRAE	600	7	287 ± 18.9	280 ± 28.3	303 ± 30.5	316 ± 42.8	36.6 ± 2.88	40.9 ± 7.1	33.4 ± 15.16	37.3 ± 15.8	21.6 ± 3.53	14.9 ± 4.33	16.6 ± 6.61	24.2 ± 18.98
Bear bile	200	7	279 ± 28.0	263 ± 28.3	279 ± 35.4	302 ± 44.8	29.6 ± 6.07	41.5 ± 12.4	31.4 ± 11.20	35.9 ± 11.0	18.6 ± 5.49	21.1 ± 13.6	17.2 ± 5.74	21.4 ± 4.96
TUDCA	20	7	308 ± 48.2	291 ± 56.7	324 ± 30.6	362 ± 30.5	32.5 ± 0.35	42.5 ± 15.9	40.5 ± 7.42	37.0 ± 4.24	19.8 ± 3.32	24.3 ± 11.8	22.3 ± 3.59	20.6 ± 1.31

^△^p < 0.05 when compared with normal group; ^△△^p < 0.01 when compared with normal group; ^*^p < 0.05 when compared with model group; ^**^p < 0.01 when compared with model group.

### The effect of drug intervention on the hepatobiliary function of rats with liver fibrosis

**Figure 2:**
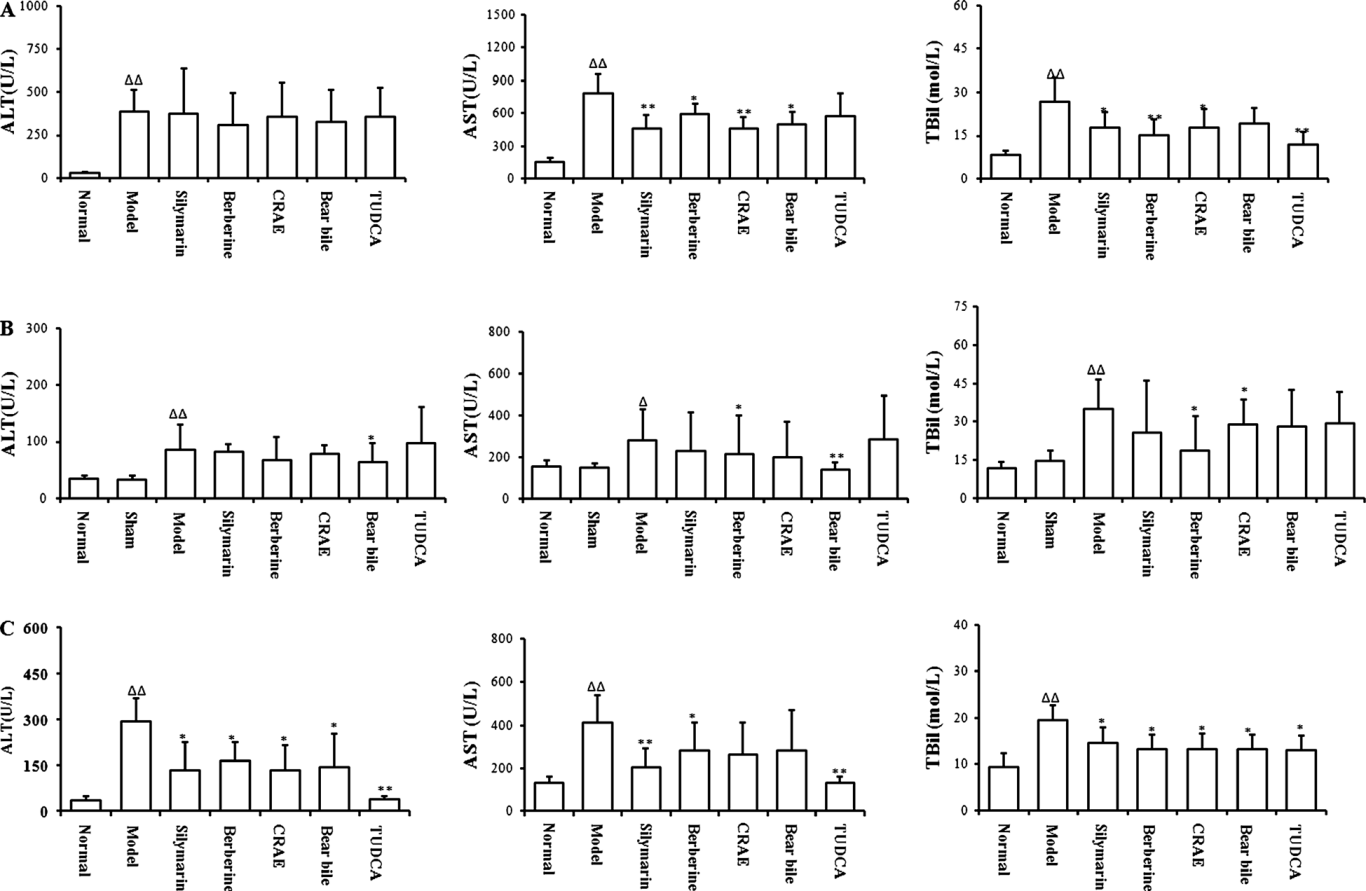
**The effect of drug intervention on serum AST, ALT and TBil level in liver fibrotic rats. **(**A**) shows AST, ALT and TBil levels in CCl_4_-induced liver fibrosis in rats. (**B**) shows AST, ALT and TBil level in bile duct ligation-induced liver fibrosis in rats. (**C**) shows AST, ALT and TBil level in alcohol induced liver fibrosis in rats. △p < 0.05, △△p < 0.01 when compared with normal group; *p < 0.05, **p < 0.01 when compared with model group.

Significant elevation of serums AST and ALT could be observed in rats with liver fibrosis induced by CCl_4_, BDL or alcohol fed, which indicated chronic liver damages in model animals (p < 0.05, Figure 2A &B&C). Increased TBil level indicated that liver fibrosis induced by CCl_4_, BDL and alcohol might also disrupted the normal structure of bile duct and block the bilirubin excretion (p < 0.05, Figure 2A &B&C). It was observed that in rats with liver fibrosis induced by CCL_4_ or BDL, drugs intervention had no effect on the elevated levels of ALT activity in serums (p > 0.05). However, significant suppression of drug intervention on serum AST activities could be found (p < 0.05, Figure 2A &B). The decrease in elevated serum AST level might indicate the protective action of either CRAE or BB on the hepatocyte. There was no significant differential action on ALT/AST activities in rats treated with CRAE or BB (p > 0.05 when comparison was made between CRAE and BB). Decreased serum TBil level was observed in rats treated with either CRAE or BB (Figure 2A &B&C). Consistent reduction of TBil level in serum could be observed in three animal models with drugs intervention. The global effective action of CRAE and BB on TBil cleavage indicated that TBil cleavage might be the more dominant mechanism in CRAE and BB’s hepatoprotective action in experimental liver fibrosis in rats.

### The effect of drug intervention on hepatic fibrosis in rats

**Figure 3:**
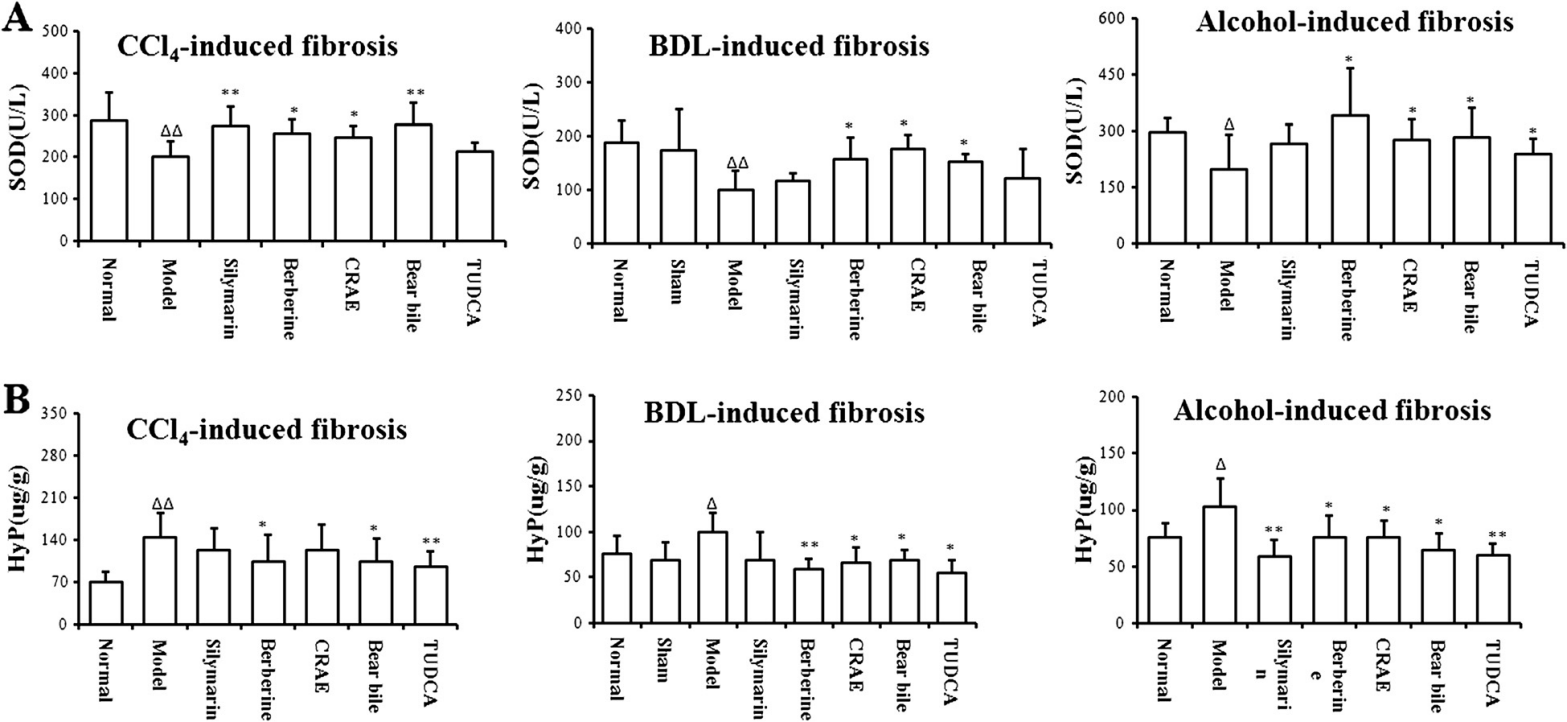
**The effect of drug intervention on the tissue HyP content and SOD activity in liver fibrotic rats. **(**A**) shows that drug intervention could reduce the HyP content in the liver of rats with fibrosis induced by CCl_4_, BDL and alcohol. (**B**) shows that drug intervention could recover the SOD activity in liver. △p < 0.05, △△p < 0.01 when compared with normal group; *p < 0.05, **p < 0.01 when compared with model group.

**Figure 4:**
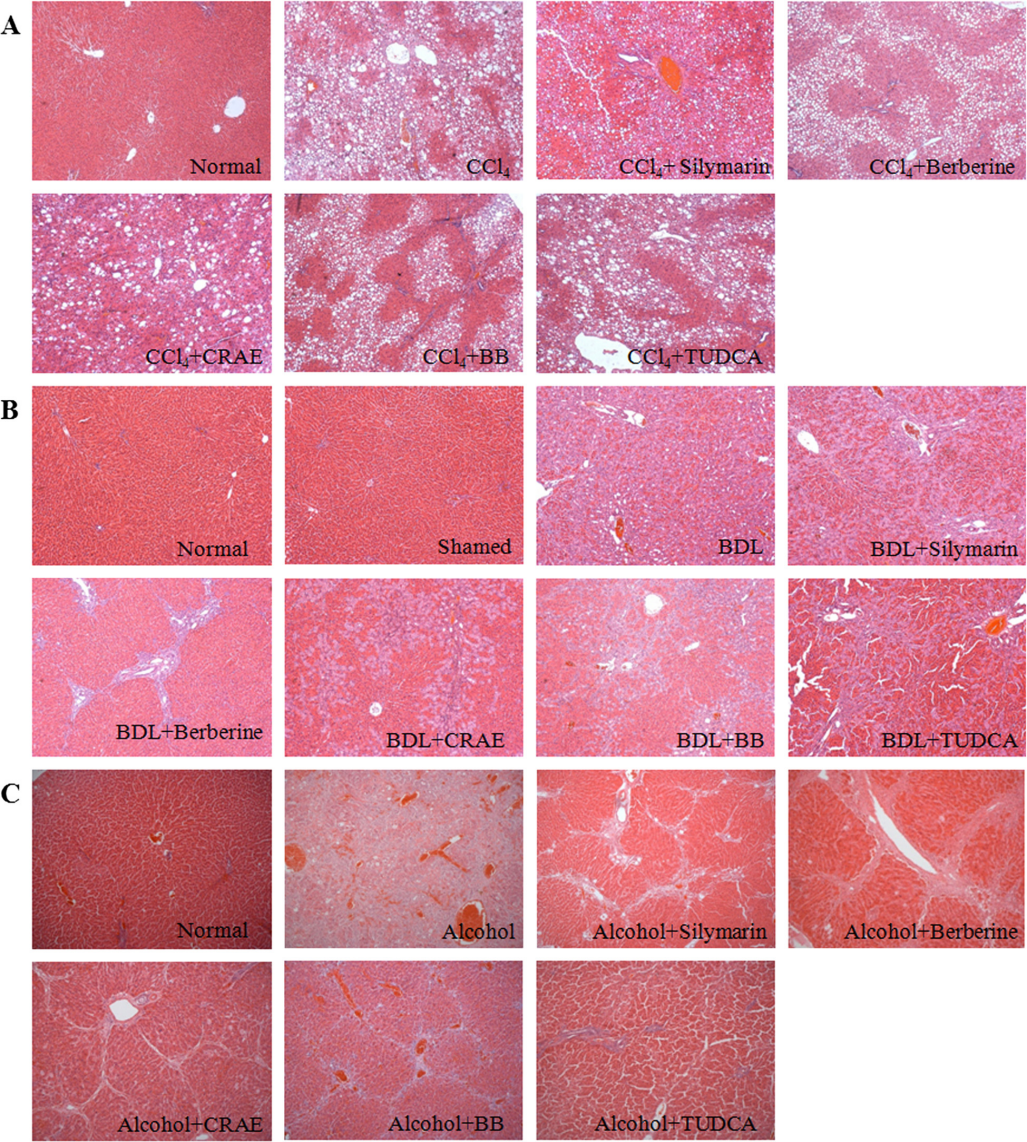
**Histological observation on the liver sections from fibrotic rats with or without drug intervention. **(**A**) shows sections in rats with liver fibrosis induced by CCl_4_. (**B**) shows sections in rats with liver fibrosis induced by bile duct-ligation. (**C**) shows sections in rats with liver fibrosis induced by alcohol. All the drugs could have potent action of preventing liver fibrosis induced by alcohol.

Significant induction on the Hyp content, the biomarker of liver fibrosis in tissue, was observed (p < 0.05). CCl_4_, BDL and alcohol could induce the up-regulation of Hyp content in liver, indicating that these interventions could successfully induce hepatic fibrosis. Drug intervention could reduce the Hyp content in modeled rats (p < 0.05). The effect of CRAE (or Berberine) is comparable with BB (or TUDCA) in treating liver fibrosis induced by CCl_4_ (p > 0.05). However, it was observed that BB (TUDCA) might exhibit better effect in inhibiting Hyp content than CRAE (or Berberine) did in hepatic fibrosis though no statistical significance could be found (p > 0.05). The results were shown in Figure 3A. Six sections from each group were selected randomly and the histological study was conducted by three individual examiners. The level of fibrosis was evaluated, and the fibrotic area within 1.5 mm^2^ of each section was measured under the light microscope. Our results showed that CCl_4_, BDL and alcohol could successfully induce fibrogenesis in rats (p < 0.05 when compared with normal group). The liver fibrosis was mildly attenuated by the presence of silymarin and TUDCA (p > 0.05). Significant therapeutic action of CRAE and BB was observed in the respective group of rats (p < 0.05). Berberine exhibited similar anti-fibrotic effect to CRAE (p > 0.05). The results were shown in Figure 4 and Tables 5, 6 and 7.

**Table 5:** **Analysis on effect of drug intervention against fibrogenesis induced by CCl**_**4**_**in rats (n = 7,**$\bar{x}\pm s$**)**

**Group**	**Sample (n)**	**Fibrosis phase**					**Fibrotic area within 1.5 mm**^**2**^**(mm**^**2**^**)**
		**S0**	**S1**	**S2**	**S3**	**S4**	
Nomral	7	7	0	0	0	0	0
Model	7	0	0	2	4	1	0.090 ± 0.021^**△△**^
Silymarin	7	0	2	3	2	0	0.067 ± 0.046
Berberine	7	0	3	2	2	0	0.041 ± 0.020^**^
CRAE	7	0	2	3	2	0	0.056 ± 0.021^*^
Bear Bile	7	0	3	3	1	0	0.048 ± 0.046^*^
TUDCA	7	0	2	3	0	2	0.065 ± 0.047

^△^p < 0.05 when compared with normal group; ^△△^p < 0.01 when compared with normal group; ^*^p < 0.05 when compared with model group; ^**^p < 0.01 when compared with model group.

**Table 6:** **Analysis on effect of drug intervention against fibrogenesis induced by bile duct-ligation in rats (n = 7,**$\bar{x}\pm s$**)**

**Group**	**Sample (n)**	**Fibrosis phase**				**Fibrotic area within 1.5 mm**^**2**^**(mm2)**
		**0**	**1**	**2**	**3**	
Normal	7	7	0	0	0	0
Shamed	7	7	0	0	0	0
Model	7	0	2	1	4	0.88 ± 0.46^△△^
Silymarin	7	0	3	2	2	0.62 ± 0.21^*^
Berberine	7	0	4	2	1	0.38 ± 0.25^**^
CRAE	7	0	4	2	1	0.55 ± 0.21^*^
Bear Bile	7	0	5	1	1	0.32 ± 0.19^**^
TUDCA	7	0	3	1	3	0.61 ± 0.32

^△^p < 0.05 when compared with normal group; ^△△^p < 0.01 when compared with normal group; ^*^p < 0.05 when compared with model group; ^**^p < 0.01 when compared with model group.

**Table 7:** **Analysis on effect of drug intervention against fibrogenesis induced by alcohol fed in rats (n = 7,**$\bar{x}\pm s$**)**

**Group**	**Samplev (n)**	**Fibrosis Phase**					**Fibrotic area within 1.5 mm2(mm2)**
		**0**	**1**	**2**	**3**	**4**	
Nomral	7	7	0	0	0	0	0
Model	7	0	0	1	4	2	0.52 ± 0.23 ^△△^
Silymarin	7	0	0	6	1	0	0.27 ± 0.10^**^
Berberine	7	0	0	5	2	0	0.34 ± 0.18^*^
CRAE	7	0	2	4	1	0	0.28 ± 0.14^**^
Bear Bile	7	0	2	4	1	0	0.26 ± 0.12^**^
TUDCA	7	1	6	0	0	0	0.14 ± 0.049^**^

^△^p < 0.05 when compared with normal group; ^△△^p < 0.01 when compared with normal group; ^*^p < 0.05 when compared with model group; ^**^p < 0.01 when compared with model group.

### The anti-oxidative action of drug intervention on liver fibrosis in rats

Significant drop of tissue SOD levels could be observed in liver fibrotic rats induced by CCl_4_, BDL and alcohol (Figure 4). The reduction of SOD levels in liver might lead to high contents of peroxides, chronic inflammation and destruction to the liver, which was the major cause of hepatic fibrosis. Recovery of SOD levels in liver was observed in rats with intervention of CRAE, berberine and BB (p < 0.05). However, the major active compound in BB, TUDCA failed to restore the SOD levels in rats with liver fibrosis (p > 0.05), which indicated that a different mechanism might be involved in the anti-fibrotic action of TUDCA (Figure 3B).

### Histological observation

Histological analysis was performed by three individual pathologists to analyze the section. Severe hepatic damage and fibrosis could be successfully induced by CCl_4_, BDL or alcohol in rats (p < 0.05). Most of the drugs exhibited potent therapeutic effect regarding the combined scores based on the examination of hepatocyte death, inflammatory cell infiltration and fibrosis (p < 0.05 in most of the cases). It was observed that both CRAE/Berberine and Bear bile/TUDCA could completely combat the liver fibrosis induced by alcohol (p < 0.05, Table 8), while in rats with hepatic fibrosis by CCl_4_ and BDL, the agents exhibited differential effects. Bear bile and its compound TUDCA exhibited better effect on chemical toxin CCl_4_-induced hepatic damage and fibrosis (p < 0.05, Table 9); and CRAE and berberine showed potent action on BDL-induced fibrosis (p < 0.05, Table 10).

**Table 8:** **Semi-quantitative histological analysis on effect of drug intervention against liver fibrosis induced by Alcohol in rats (n = 7,**$\bar{x}\pm s$**)**

**Group**	**Hepatocyte death**	**Inflammatory cell infiltration**	**Fibrosis**	**Combined score**
Normal	0.3 ± 0.2	3.0 ± 0.1	0.1 ± 0.1	0.3 ± 0.1
Model	4.1 ± 0.7^△△^	3.3 ± 0.6^△△^	4.3 ± 0.7^△△^	4.0 ± 0.6^△△^
Sylimarin	1.5 ± 0.7^**^	1.6 ± 0.5^**^	3.2 ± 0.5	2.6 ± 0.5^*^
BBR	1.3 ± 0.3^**^	1.4 ± 0.5^**^	1.8 ± 0.3^**^	2.2 ± 0.5^**^
CRAE	1.5 ± 0.7^**^	1.3 ± 0.6^**^	2.2 ± 0.5^**^	2.5 ± 0.4^*^
BB	1.6 ± 0.5^**^	1.3 ± 30.5^**^	1.2 ± 0.4^**^	2.0 ± 0.4^**^
TUDCA	0.9 ± 0.4^**^	0.7 ± 0.3^**^	0.8 ± 0.3^**^	1.0 ± 0.4^**^

^△^p < 0.05 when compared with normal group; ^△△^p < 0.01 when compared with normal group; ^*^p < 0.05 when compared with model group; ^**^p < 0.01 when compared with model group.

**Table 9:** **Semi-quantitative histological analysis on effect of drug intervention against liver fibrosis induced by CCl**_**4**_**in rats(n = 7,**$\bar{x}\pm s$**)**

**Group**	**Hepatocyte death**	**Inflammatory cell infiltration**	**Fibrosis**	**Combined score**
Normal	0.8 ± 0.3	0.3 ± 0.2	0.3 ± 0.2	0.5 ± 0.1
Model	5.6 ± 0.9^△△^	3.9 ± 0.4^△△^	3.3 ± 0.6^△△^	4.8 ± 0.7^△△^
Sylimarin	3.9 ± 0.2^*^	2.8 ± 0.6^*^	2.3 ± 0.6	3.0 ± 0.9^*^
Berberine	3.5 ± 0.4^*^	3.1 ± 0.4	2.0 ± 0.5^*^	3.0 ± 0.6^*^
CRAE	3.8 ± 0.4^*^	3.0 ± 0.3^*^	2.1 ± 0.4^*^	3.2 ± 0.5^*^
Bear Bile	3.1 ± 0.3^**^	2.7 ± 0.7^*^	2.4 ± 0.5	2.8 ± 0.5^**^
TUDCA	4.3 ± 0.7	3.5 ± 0.4	2.8 ± 0.2	4.1 ± 0.4

^△^p < 0.05 when compared with normal group; ^△△^p < 0.01 when compared with normal group; ^*^p < 0.05 when compared with model group; ^**^p < 0.01 when compared with model group.

**Table 10:** **Semi-quantitative histological analysis on effect of drug intervention against liver fibrosis induced by BDL in rats(n = 7,**$\bar{x}\pm s$**)**

**Group**	**Hepatocyte death**	**Inflammatory cell infiltration**	**Fibrosis**	**Combined score**
Normal	0.5 ± 0.3	0.3 ± 0.3	0.5 ± 0.1	0.5 ± 0.3
Shamed	0.8 ± 0.3	0.7 ± 0.4	0.5 ± 0.3	0.8 ± 0.3
Model	4.7 ± 0.8^△△^	4.7 ± 0.4^△△^	5.5 ± 0.7^△△^	4.8 ± 0.6^△△^
Sylimarin	3.5 ± 0.3^*^	4.4 ± 0.9	4.5 ± 0.6	4.2 ± 0.5
Berberine	2.4 ± 0.5^**^	2.3 ± 0.7^**^	2.7 ± 0.7^**^	2.5 ± 0.6^**^
CRAE	2.7 ± 0.4^**^	3.4 ± 0.9^*^	3.3 ± 0.3^**^	3.0 ± 0.3^**^
Bear Bile	3.9 ± 0.1	3.5 ± 0.4^**^	3.3 ± 0.6^**^	3.8 ± 0.4^*^
TUDCA	2.8 ± 0.3^**^	3.7 ± 0.2^**^	3.6 ± 0.2^**^	3.4 ± 0.2^**^

^△^p < 0.05 when compared with normal group; ^△△^p < 0.01 when compared with normal group; ^*^p < 0.05 when compared with model group; ^**^p < 0.01 when compared with model group.

## Discussion

Bear bile has been used as a remedy in Chinese medicine for more than thousands years. Bear bile was also widely used in the Chinese Medicine formulae by practitioners during their clinical practice. According to the China’s State Pharmacopoeia (1995 edition), 28 medicines contained bear bile, indicating its frequent use in Chinese medicine [14]. However, the use of bear bile has received various criticisms in the recent years. From the ecological standpoint, obtaining bile from wild or farmed bears undoubtedly endangers the species to the brink of extinction. The establishment of unethical bear farming also brings about pain and unexpected diseases to the animal. Most of all, there are many who are worried that the use of animal products brings about cross-contamination of infectious diseases to the human population [15]. China’s State Pharmacopoeia, starting with the 2010 edition, no longer includes any medicine whose ingredients are derived from endangered wild animals including bear bile. A suitable alternative for bear bile is of paramount concern.

Extensive studies have indicated that bear bile in Chinese Medicine may be possible to have some alternatives in herbal or chemical remedies, or products from domestic animals [2,16]. Some studies have found that the similarities pharmacological actions on rabbit bile and bear bile [17]. It was also reported that both bear and pig bile solutions exert similar anti-inflammatory, anti-convulsive, and analgesic action [18]. TUDCA, the characteristic composition of bear bile, has been successfully synthesized and used as a commercial product for the treatment of liver diseases as well as its unconjugated form, Ursodeoxycholic acid (UDCA) [19]. A recent study reported that Scutellaria baicalensis Georgi, Huangqin in Chinese, exhibited anti-inflammatory action which indicated its potential as an alternative of bear bile [18]. Bear Bile is a traditional Chinese Medicine material used in treating various different diseases by relieving toxin, stopping endogenous win to arrest convulsion and clearing liver fire to improve eyesight [2]. Bear bile powder as an individual product has been used to treat liver diseases in China for many years. Its active compound TUDCA was developed to protect the liver in clinics though some adverse effects have been reported [20]. Coptidis Rhizoma belongs to the same category with bear bile according to Chinese Medicine theories, and has been shown to benefit eye function in some ancient Chinese medical books [2]. The property of Coptidis Rhizoma in treating eye diseases is similar to that of bear bile, and studies in our group have exhibited the beneficial effect of Coptidis Rhizoma on acute and chronic liver injury [5,6]. These observations supported Coptidis Rhizoma as an alternative for bear bile as a medication in treating liver diseases. However, Chinese Medicine is a very complicated medical system. The medication used by Chinese Medicine practitioners, called Chinese Medicines in Hong Kong, is composed of herbs, animal products and minerals. Each Chinese Medicine medication is a mixture with multiple components, and is considered to have various therapeutic effects against different diseases. In general, Chinese Medicine practitioners use medical composite formulae in their clinical practices, both Bear Bile and Coptidis Rhizoma could be used in formulae or as a single medication, but it is not reasonable to simply replace bear bile with Coptidis Rhizoma in Chinese Medicine formulae. Based on our current observations, we could only draw a conclusion that Coptidis Rhizoma has comparable anti-fibrotic action with bear bile, and it might be applicable to use Coptidis Rhizoma as an alternative when bear bile is applied in the form of single medication to treat liver fibrosis. Whether Coptidis Rhizoma could be use in treating other diseases as bear bile does needs further investigation, and more comprehensive studies should be conducted before concluding that Coptidis Rhizoma could replace bear bile in clinical practice.

**Figure 5:**
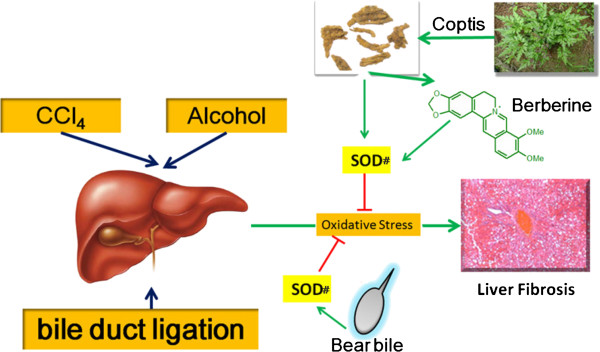
Illustrated overview for this study.

As a principal active compound in Coptidis Rhizoma, berberine has lots of pharmacological properties, including anti-microbial [21], anti-inflammatory [22] anti-hypertensive [23], anti-diabetic [24], anti-hyperlipidemic activities [25]. As a commercial product, berberine chloride is conventionally used for the treatment of bacterial diarrhea, intestinal parasitic infections, and ocular trachoma infections. Some recent studies reported the anti-fibrotic properties of berberine, indicated its potential for the treatment of chronic liver diseases. It was reported that berberine could attenuate the liver fibrosis induced by multiple hepatotoxic factors in rats [26]. *In vitro* study revealed that berberine inhibited the hepatic stellate cell proliferation via arresting the cell cycle at G1 phase [27]. In our study, we found that berberine selectively increased the activity of SOD, whose function was to reduce lipid hydroperoxides to their corresponding alcohols and to reduce free hydrogen peroxide to water [28]. This indicated that berberine protected liver from oxidative damage and therefore reduced liver fibrosis. In our previous publications, we have investigated the effect of Coptidis Rzhioma and berberine on acute and chronic liver injury [5-7]. Coptidis Rzhioma and berberine exhibited protective effect on acute and chronic liver injury in animal models. And we also studied the underlying mechanism *in vitro* in our previous study [6], we found that Coptidis Rzhioma and berberine could up-regulate SOD to scavenge oxidative stress, which activates Erk1/2 and induces apoptosis in hepatocytes. As constitutive and chronic injury has been considered as the major cause of liver fibrosis, we continued to focus on the effect of SOD in this study. We also detected some anti-oxidative related molecules and enzymes (Data not shown). However, we found that only SOD was elevated by Coptidis Rzhioma and Bear Bile. So we drew a conclusion that SOD should at least take involvement in the anti-fibrosis of Coptis and Bear Bile. We could not conclude that it is the unique mechanism since Chinese Medicine should be a complex and multi-target system. However, from our results, SOD up-regulation should make it count. The possible mechanism involved is as shown in Figure 5. Moreover, reduced TBil levels in serum indicated that berberine was able to excrete the bilious product and prevent the hepatocyte damage induced by bilious product accumulation. These observations draw light on berberine’s potential as a therapeutic agent for both cholestatic and non-cholestatic liver fibrosis and can be also alternative agent to bear bile.

Our research demonstrated that CRAE and berberine had better effects than bear bile and TUDCA in liver fibrosis animal models. In addition, CRAE is under the same category of bear bile in Chinese Medicines, thus CRAE and berberine as the substitutes for bear bile for liver fibrosis are supported by the traditional reason and scientific evidences.

## Conclusion

A comparative study on the hepatoprotective action of CRAE and bear bile on experimental liver fibrosis in rats was conducted. Berberine and TUDCA, as the main component of CRAE and bear bile respectively, were also included in this study to evaluate their anti-fibrosis activity. We also observed that CRAE, berberine, and bear bile exerted anti-fibrotic properties on the liver of rats. CRAE and berberine reduced the peroxidative stress in liver through increasing the SOD enzyme activity. CRAE and berberine were capable of facilitating harmful bilious product excreted by liver and protecting hepatocyte from cholestatic damage. The anti-fibrotic action of CRAE and berberine was comparable to bear bile on the all three experimental animal models. CRAE and its major component berberine had the most potential to replace bear bile for the treatment of liver diseases.

## Competing interests

The authors declare no competing of interests.

## Authors's contributions

YF designed the experiment, analyzed the data and drafted the manuscript; NW conducted the experiment and helped draft the manuscript; OYC XW and WS conducted the experiment; FC and YT analyzed the data. All authors read and approved the final manuscript.

## Pre-publication history

The pre-publication history for this paper can be accessed here:

http://www.biomedcentral.com/1472-6882/12/239/prepub
